# Social Integration, Functional Independence, and Depressive Symptoms: Long-Term Effects After TBI in Older Adults

**DOI:** 10.1093/geroni/igaf122.1325

**Published:** 2025-12-31

**Authors:** Wonkyung (Kelly) Jung, Dabin Hwang, Janiece Taylor

**Affiliations:** Boston College, Chestnut Hill, Massachusetts, United States; Boston College William F. Connell School of Nursing, Chestnut Hill, Massachusetts, United States; Johns Hopkins University, Baltimore, Maryland, United States

## Abstract

Traumatic Brain Injury (TBI) is a leading cause of disability worldwide, particularly among older adults. The resulting disabilities can significantly affect social integration, functional independence, and contribute to depressive symptoms. Addressing these factors is vital for promoting healthy aging in older individuals post-TBI. However, research on the interplay between social integration, functional independence, and depressive symptoms in this population is limited. This study examines the reciprocal associations between these factors over 5 years post-injury. We conducted a secondary data analysis using the Traumatic Brain Injury Model Systems National Database (TBIMS-NDB). Structural equation modeling (SEM) was used to explore the causality and directionality of the relationships between social integration, functional independence, and depressive symptoms in older adults post-TBI. A cross-lagged analysis (n = 2101) assessed longitudinal changes in these variables at 1-, 2-, and 5-years post-injury, adjusting for covariates. Social integration was associated with improved functional independence but did not predict depressive symptoms. Greater functional independence was linked to better social integration and fewer depressive symptoms. Higher levels of depressive symptoms were associated with poorer functional independence. Cross-lagged relationships between social integration and functional independence, as well as between functional independence and depressive symptoms, suggest reciprocal effects that accumulate over time. These findings emphasize the interconnectedness of these factors and the importance of addressing them in interventions aimed at improving long-term well-being in older adults post-TBI.

